# Wnt/β-Catenin signaling pathway in hepatocellular carcinoma: pathogenic role and therapeutic target

**DOI:** 10.3389/fonc.2024.1367364

**Published:** 2024-04-02

**Authors:** Zekun Zhao, Tenglu Cui, Fengxian Wei, Zhiming Zhou, Yuan Sun, Chaofeng Gao, Xiaodong Xu, Huihan Zhang

**Affiliations:** ^1^ The Second Hospital of Lanzhou University, Lanzhou, China; ^2^ The Second General Surgery Department, The Second Hospital of Lanzhou University, Lanzhou, China; ^3^ The Radiotherapy Department, The Second Hospital of Lanzhou University, Lanzhou, China

**Keywords:** Wnt pathway, hepatocellular carcinoma, tumorigenesis, progression, therapy resistance, mechanism, therapy

## Abstract

Hepatocellular carcinoma (HCC) is the most common primary malignant liver tumor and one of the leading causes of cancer-related deaths worldwide. The Wnt/β-Catenin signaling pathway is a highly conserved pathway involved in several biological processes, including the improper regulation that leads to the tumorigenesis and progression of cancer. New studies have found that abnormal activation of the Wnt/β-Catenin signaling pathway is a major cause of HCC tumorigenesis, progression, and resistance to therapy. New perspectives and approaches to treating HCC will arise from understanding this pathway. This article offers a thorough analysis of the Wnt/β-Catenin signaling pathway’s function and its therapeutic implications in HCC.

## 1 Introduction

Hepatocellular carcinoma (HCC) is the most prevalent primary malignant tumor of the liver and accounts for over 850,000 deaths from cancer-related causes globally each year (1, 2). In recent years, its morbidity and mortality have gradually increased (3). Despite significant advancements in HCC research, the prognosis of HCC is still terrible because the early symptoms of patients are not obvious and the disease has often progressed to the advanced stage when diagnosed (4). Radical hepatectomy is currently the first-line treatment for patients with HCC, but it is limited to patients with early-stage HCC who have good liver function, small masses, and no vascular invasion. However, due to its extremely high postoperative recurrence and metastasis rates, the 5-year survival rate of patients is only 19% (5). Liver transplantation offers another potential therapeutic option for patients with HCC, as it can remove the tumor while preventing the occurrence of postoperative liver stiffness in patients. However, when performing liver transplantation, it is extremely important to evaluate the patient’s stage, and the presence of extrahepatic metastases or vascular infiltration may lead to treatment failure. Moreover, due to the shortage of organ donors and the complexity of the procedure, its clinical implementation remains difficult. In addition, for some patients with intermediate to advanced HCC, chemotherapy offers a new option. First-line chemotherapy regimens based on sorafenib and lenvatinib are currently the treatment of choice for patients with advanced HCC, but the survival of most patients does not improve due to the resistance of HCC to chemotherapy and the development of cirrhosis in patients in the later stages of treatment. Furthermore, considering the complexity of HCC pathogenesis, targeted therapies may provide a more effective treatment option for HCC patients, which can specifically target key signaling pathways dysregulated during HCC pathogenesis through small molecules or monoclonal antibodies to achieve the goal of inhibiting tumor cell growth and inducing apoptosis. However, despite clinical trials of most drugs, there are still fewer targeted drugs approved for clinical use in HCC (6). Therefore, it is crucial to investigate the regulatory mechanisms behind the pathogenic process of HCC to identify novel biomarkers and therapeutic targets for the early diagnosis and treatment of HCC patients.

A highly conserved signaling pathway, the Wnt/β-Catenin signaling pathway, is also referred to as the typical Wnt signaling pathway. It is essential for liver development, metabolic zonation, and regeneration (7). It has been reported that abnormal activation of this pathway is a major carcinogen in liver cancer, and gene mutations encoding components of the pathway have been found in more than 80% of liver cancer patients (8). Mutations in the CTNNB1 gene, which encodes β-catenin, an essential constituent of this system, have been identified in various types of tumors, with HCC exhibiting the greatest frequency of such mutations (9). Recent studies have demonstrated that aberrant activation of the Wnt/β-Catenin signaling pathway is a significant contributor to the tumorigenesis, progression, and therapy resistance of HCC. Inhibiting this pathway holds promise as a hopeful therapeutic approach for HCC (10). In this review, we discuss the physiological role of the Wnt/β-Catenin signaling pathway in human liver and its mechanism in promoting the tumorigenesis, progression, and therapy resistance of HCC. In addition, we also explored the potential significance of this pathway in targeted therapy of HCC. In order to better understand the mechanism of action of this pathway in HCC and provide new directions for HCC-targeted treatment.

## 2 Overview of Wnt signaling pathway

The Wnt signaling pathway is an important signaling pathway for maintaining homeostasis from embryonic development to adulthood and plays a vital role in numerous biological processes (11). Typically, the canonical and non-canonical pathways make up the two kinds of Wnt pathways. Among them, the Wnt/β-Catenin signaling pathway is the canonical pathway, while the non-canonical pathway mainly includes the Wnt/PCP (Planar cell polarity) and Wnt/Ca2+ signaling pathways (12, 13).

### 2.1 Canonical Wnt/β-Catenin signaling pathway

As an important transcription factor in the Wnt/β-Catenin signaling pathway, stabilization and nuclear translocation of β-Catenin mediated by Wnt ligands is a key mechanism of this pathway (14, 15). Without Wnt ligands, β-Catenin is phosphorylated and degraded by a complex consisting of Axin, Glycogen synthase kinase 3β (GSK-3β), Casein kinase I (CKI) and Adenomatous polyposis (APC). In this process, Axin serves as a scaffold protein, with CKI and GSK-3β sequentially phosphorylating β-Catenin, and APC-mediated recognition and eventual degradation of phosphorylated β-Catenin by the E3 ubiquitin ligase β-TrCP, which maintains the stability of intracellular cytoplasmic levels of β-Catenin and prevents the expression of target genes of the pathway (16–20).

**Figure 1:**
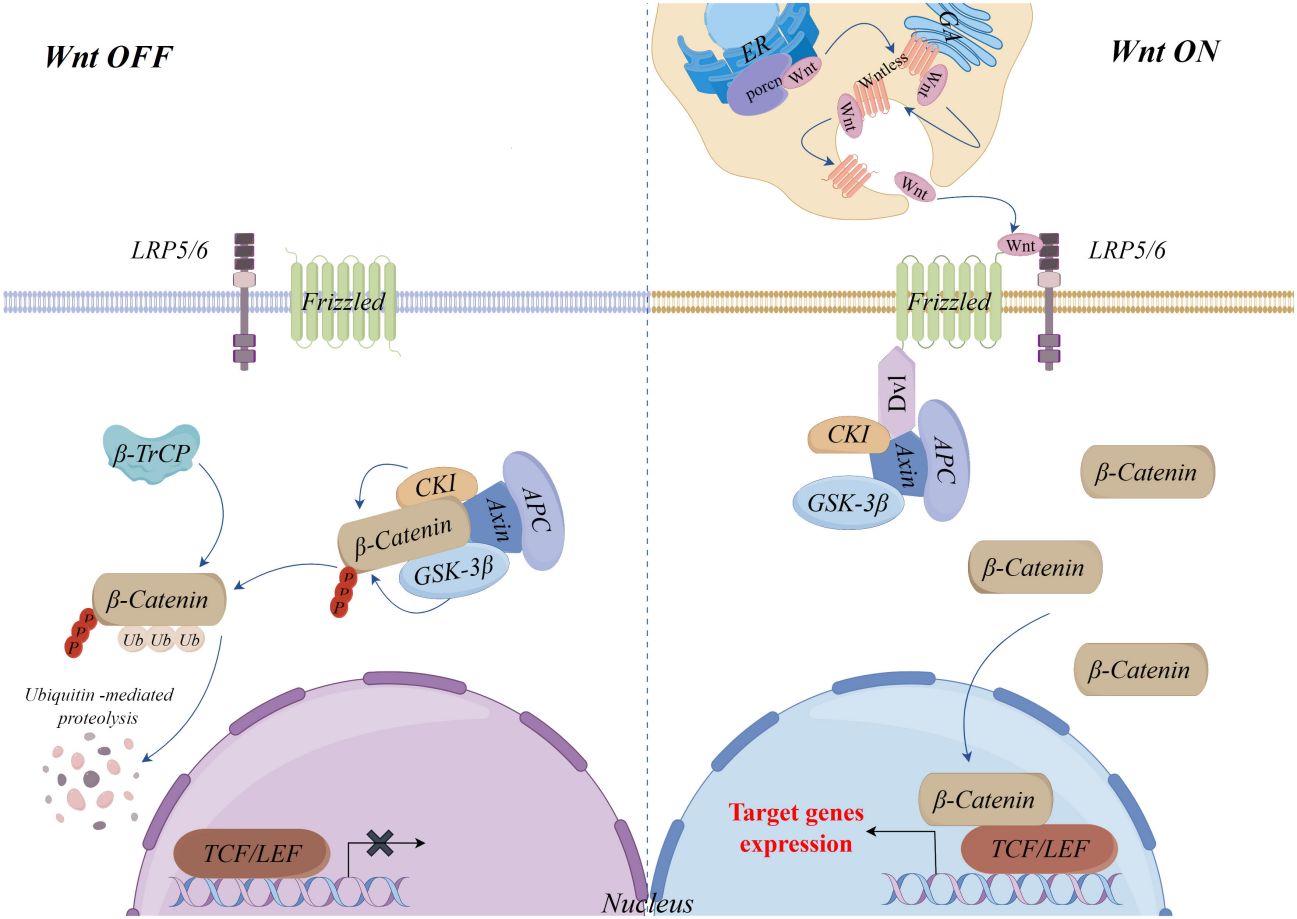
Wnt/β-Catenin signaling pathway.

Wnt ligands (Wnts) are secreted glycoproteins and there are 19 known Wnts (21). Before secretion, Wnts must be glycosylated and palmitoylated by the endoplasmic reticulum’s Porcupine O-acyltransferase (PORCN), a process that is essential for Wnts secretion and function (22). Additionally, the secretion of Wnts requires a carrier protein, Wntless, a multi-pass transmembrane protein. Wnts need to bind Wntless and are transported to the cell membrane for secretion via the Golgi vesicular system (23, 24). Once secreted, Wnts bind to the transmembrane Frizzled (FZD) receptors and coreceptors low-density lipoprotein receptor-related proteins 5/6 (LRP5/6), recruiting cytoplasmic Disheveled (Dvl) proteins, leading to the phosphorylation of LRP5/6 and attract the Axin complex to the plasma membrane. Through this mechanism, the Axin complex is unable to phosphorylate and destroy β-Catenin, which allows it to build up in the cytoplasm and then go into the nucleus. There, it binds to T-cell factor (TCF)/lymphoid enhancer factor (LEF) family transcription factors and coactivators, initiating the transcriptional expression of downstream target genes (25–27) (**Figure 1**).

### 2.2 Non−canonical Wnt signaling pathway

In addition to the canonical Wnt/β-Catenin signaling pathway, non-canonical Wnt pathways also play an important role, including Wnt/PCP and Wnt/Ca^2+^ signaling pathways. Studies have shown that non-canonical Wnt pathways are not only involved in the regulation of various cellular functions but also may be involved in the occurrence and progression of cancer when activated or inhibited abnormally (28). In the Wnt/PCP signaling pathway, Wnts bind to FZD receptors on the cell surface and further activate Jun N-terminal kinases (JNKs) by recruiting Dvl to activate the RAC and its downstream mitogen-activating protein 3 kinases (MAP3Ks) and mitogen-activated protein 2 kinases (MAP2Ks). In addition, Dvl can also activate its downstream Ras homolog gene-family members A (RhoA) and Rho-associated kinases (ROCK) through the Dvl-associated activator of morphogenesis 1 (Daam1), where RhoA can also activate JNK. This process regulates the cytoskeleton and initiates the expression of downstream target genes to facilitate cell motility (29, 30).

**Figure 2:**
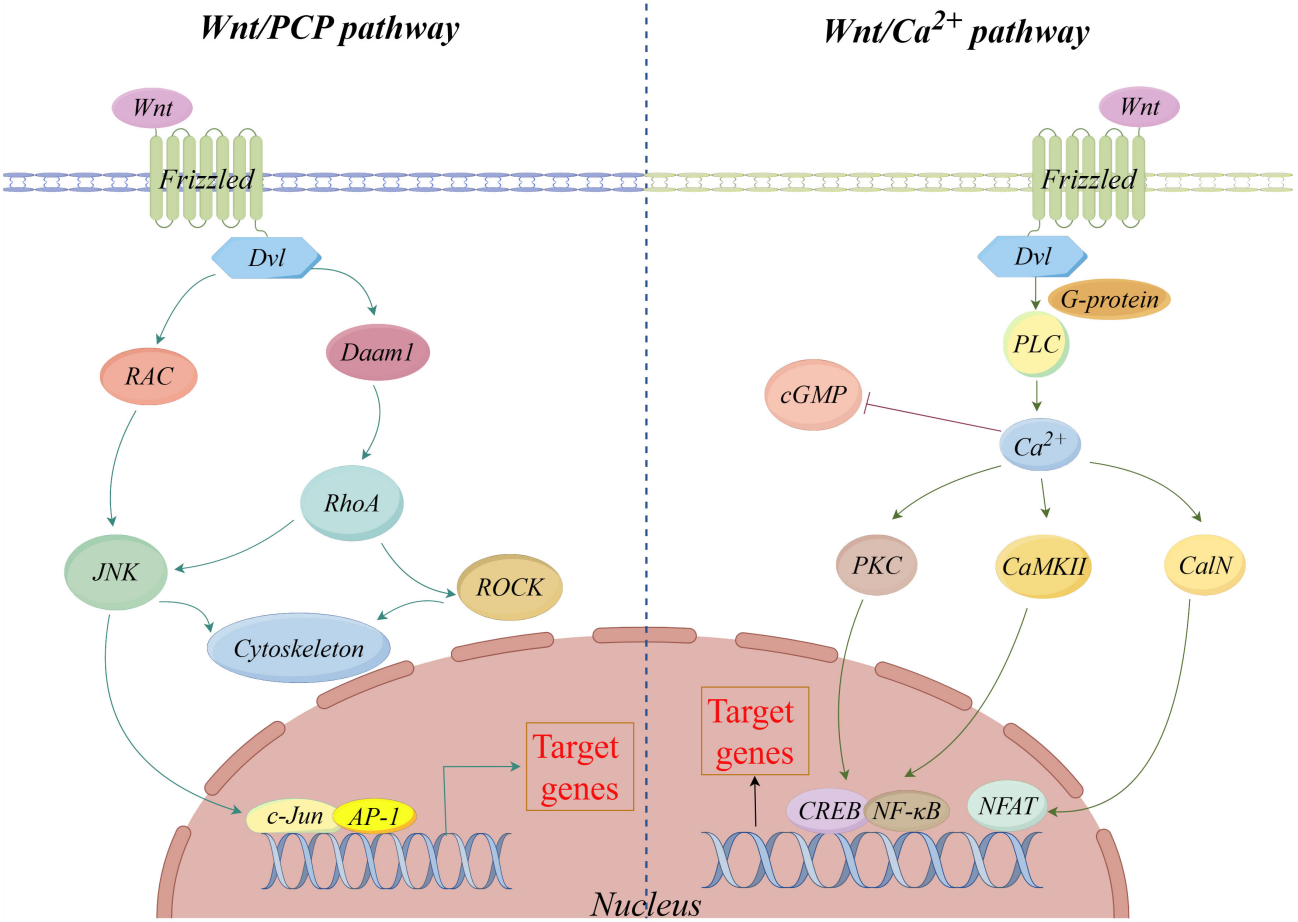
Non−canonical Wnt signaling pathway.

In the Wnt/Ca^2+^ signaling pathway, the interaction of Wnts with the FZD receptor leads to the activation of homotrimeric G proteins, which in turn activate phospholipase C (PLC). Activation of PLC leads to an increase in intracellular Ca^2+^, which inhibits the expression of cyclic guanosine monophosphate (cGMP) and promotes the activation of calmodulin-dependent protein kinase II (CaMKII) or calcineurin (CalN) and protein kinase C (PKC). Subsequently, cAMP response element binding protein (CREB) and Nuclear factor kappa-B (NF-κB) are activated by CaMKII and PKC, and the Nuclear factor of activated T cells (NFAT) in the cytoplasm is activated and dephosphorylated by CalN. Finally, CREB, NF-κB, and NFAT translocated into the nucleus and initiated the expression of target genes downstream of the Wnt/Ca^2+^ signaling pathway (13, 31, 32) (**Figure 2**).

## 3 Physiological role of the Wnt/β-catenin signaling pathway in the liver

### 3.1 Liver development

During different stages of embryonic development, the Wnt/β-catenin signaling pathway does not always promote liver development. In the gastrula and early somite formation stages, the endoderm along the anterior-posterior axis is divided into foregut, midgut, and hindgut, with the liver ultimately originating from the foregut (33). Homeobox (HHEX) gene, one of the earliest foregut markers, can induce the production of downstream transcription factors HHEX and Forkhead Box A2 (FOXA2), promoting foregut fate and future liver development (34). The Wnt/β-catenin signaling pathway is indispensable in the initial development of the hindgut. Wnts, Fibroblast Growth Factor 4 (FGF4), and Bone Morphogenetic Proteins (BMP) from the adjacent midgut endoderm promote hindgut development while also inhibiting foregut development by suppressing HHEX and FOXA2 expression (35, 36). Thus, there is a Wnt inhibitor, secreted FZD-related protein 5 (SFRP5), in the foregut endoderm, which inhibits this pathway conductance, thereby maintaining foregut features and promoting liver development (37, 38).

The activation of the Wnt/β-catenin signaling pathway is as crucial for liver specification in later development as is the repression of this pathway during early foregut development. Liver specification occurs around embryonic day 8.5, triggered by BMP signaling from mesenchymal cells of the septum transversum and FGF from the cardiac mesoderm, under the influence of transcription factors Hepatocyte Nuclear Factor-1β (HNF-1β), Forkhead box A1 (FOXA1), FOXA2, and GATA Binding Protein 4 (GATA4) (23, 39, 40). Research indicates that Wnt2b is essential for the induction of liver specialization, with reduced expression of liver-specific genes HHEX and Prospero homeobox 1 (PROX1), as well as transient deletion of liver specialization, observed in zebrafish embryos lacking Wnt2bb (Wnt2b homolog) (41). Furthermore, as liver specification occurs, liver buds consisting of hepatoblasts begin to develop, accompanied by hepatoblast proliferation (42). Wnts, FGF, and hepatocyte growth factor (HGF) have been shown to be involved in this process, which can promote the proliferation of hepatoblast by activating this pathway (43–45). Additionally, this pathway is equally significant in postnatal liver growth. It has been shown that mice with hepatic β-catenin-specific deletion or suppressed Wnt/β-catenin signaling show a notable reduction in liver weight relative to body weight (46, 47). The opposite was true in liver-specific non-mutated β-catenin-overexpressing transgenic mice (48).

### 3.2 Metabolic zonation of liver

As the smallest structural unit in the liver, hepatic lobules can be divided into three zones based on the different metabolic functions of hepatocytes at different locations in the liver lobules: the hepatocytes adjacent to the portal vein triad constitute Zone 1, the hepatocytes adjacent to the central vein constitute Zone 3, and the hepatocytes in between constitute Zone 2 (49).

Benhamouche and colleagues were the first to report the significant function of the Wnt/β-catenin signaling pathway in guiding liver metabolic zonation and proposed the concept of APC as the “zonal guardian” gene of the liver. Their research indicated high expression of APC in periportal areas without β-catenin activation, while an absence of APC expression was found in pericentral areas with β-catenin activation (50). This elucidates the distinct manifestation of this pathway targets in the pericentral areas, including Axin2, GS (Glutamine synthetase), and cytochrome P450 enzymes (CYP2E1 and CYP1A2). Inhibition of this pathway may lead to impairment of metabolic zonation of the liver. Recent studies have found that either liver-specific LRP5/6 deletion or LRP4/5 deletion results in loss of liver metabolic regions (47, 51). Additionally, mice with liver-specific β-catenin deletion also have liver metabolic zonation disorders and cause the liver to exhibit a periportal phenotype overall (52). Whereas β-catenin accumulation caused by APC-specific inactivation exhibits an overall pericentral phenotype (50). These results indicate that the Wnt/β-catenin signaling pathway is important for liver metabolic zonation and has distinct effects on the expression of genetic programs in the periportal and pericentral regions.

### 3.3 Liver regeneration

Liver regeneration is crucial for maintaining liver homeostasis and restoring the size and function of the damaged liver. Hepatocytes, as the main contributors to liver regeneration, mediate this process through their proliferation (53–57). Monga and colleagues found that in a rat model subjected to partial hepatectomy (PHx), β-catenin increased quickly in the first five minutes and subsequently moved into the nucleus (58). Similarly, Apet and colleagues found that β-catenin dramatically rose 1 to 6 hours after acetaminophen injection in mice with acute liver failure produced by the drug, and then increased again 24 hours later. Moreover, the expression of Wnt/β-catenin signaling pathway targets GS and cyclin-D1 (Cyclin-D1) also increased during these periods (59). These findings imply that liver regeneration may be significantly aided by this pathway.

Subsequent experiments further confirmed the importance of this pathway in liver regeneration. The study found that after partial hepatectomy (PHx) in a β-catenin-specific knockout mouse model, the liver cell proliferation of mice in the experimental group was lower than that of the control group, and the liver weight/body weight ratio was significantly lower than that of the control group (60). This finding aligns with results from two other studies (46, 61). In contrast, a transgenic mouse model overexpressing β-catenin showed a significant increase in hepatocyte proliferation after receiving PHx. In addition, compared with the control group, exogenous activation of the Wnt/β-catenin signaling pathway in mice by the Wnt-1 gene can also increase the proliferation of liver cells (62). Furthermore, recent studies have shown that macrophages can regulate this pathway, upregulating important metabolic functions of non-proliferating hepatocytes in the compensatory phase of liver regeneration following acute liver injury, thereby preserving fundamental physiological functions of the liver (63). The above studies show that the Wnt/β-catenin signaling pathway is essential for the regeneration of the liver. Moreover, exogenous modification aimed at stimulating this pathway could potentially serve as a therapeutic agent to promote liver regeneration.

## 4 The role of the Wnt/β-Catenin signaling pathway in the tumorigenesis and progression of HCC

### 4.1 Wnt/β-Catenin signaling pathway and HCC tumorigenesis

Aberrant activation of the Wnt/β-catenin signaling pathway is one of the main driving factors in the tumorigenesis of HCC and is widely present in HCC patients, with their relationship being well established. Mutations in the CTNNB1 gene encoding β-catenin in Exon3 are the most common activation mechanism of this pathway (64). Research indicates that CTNNB1 gene mutations are present in about 20%-40% of HCC cases, and this mutation occurs more frequently in HCC cases related to Hepatitis C virus (HCV) infection than in HCC cases related to Hepatitis B virus (HBV) infection (65, 66). Second, loss of function or mutation of Axin, GSK-3β, and APC as members of the Axin complex can likewise activate the pathway and exert oncogenic effects. Axin1 mutations have been reported to account for approximately 3%-16% of all HCC cases, and Axin2 for approximately 3% (67). Additionally, studies have revealed that the proportions of phosphorylated GSK-3β and overexpressed β-catenin in HCC tissues are 52.2% and 56.5%, respectively, higher than in surrounding normal tissues. It is crucial to note that none of the HCC patients who exhibited phosphorylated GSK-3β possessed CTNNB1 gene mutations (68). Consistently, APC serves as one of the members of the Axin complex, and targeted inactivation of the hepatic APC gene similarly leads to overexpression of β-catenin and promotes HCC tumorigenesis (69). However, it’s noteworthy that in 40-60% of HCC cases, there are no mutations in CTNNB1, Axin1, or Axin2 (65). Based on this, another variation that controls the pathway was found in a recent genome-wide association study (GWAS) that specifically targeted alcohol-related HCC: the WNT3A-WNT9A gene variants are specifically linked to the occurrence of HCC in alcoholic liver disease patients (70, 71).

In the Wnt/β-catenin signaling pathway, 19 known Wnts that function by binding to one or more of the 10 types of FZD receptors (72). Secreted frizzled-related proteins (SFRPs) are antagonists of this pathway that bind Wnts, downregulate their ability to bind and activate FZD receptors, and inhibit this pathway (73). Studies have shown that in contrast to a normal liver, there is an upregulation of FZD3/6/7 and Wnt3/4/5a expression in 95% of HCC and 68% of the surrounding tumor tissues, along with downregulation of sFRP1/5, which accumulates progressively with tumor advancement and the severity of fibrosis in surrounding tissues (72). Among them, Wnt3 and FZD7 have been shown to activate this pathway through their interaction (74). Additionally, other studies have found that methylation of SFRP family genes is not only widely present in HCC tissues, but also in HBV- or HCV-related chronic hepatitis and cirrhosis tissues. It can lead to the down-regulation of SFRPs expression, which activates this pathway and may participate in promoting the occurrence of HCC. These events are considered early events in the tumorigenesis of HCC (75, 76).

In addition to the aforementioned mechanisms, activation of the Wnt/β-catenin signaling pathway involves various other mechanisms. It has been shown that TGF-β-dependent activation, and Receptor tyrosine kinase (RTK) activation in fibrotic laminar HCC are involved in this process (77, 78). However, the mechanisms of these two types of activation remain unclear. Epidermal Growth Factor (EGFR), a type of RTK, has been reported to be transcriptionally upregulated or aberrantly expressed in multiple cancers. Studies indicate that EGFR participates in regulating TCF-dependent β-catenin transcriptional activity in HCC through kinase-independent mechanisms, thereby participating in the regulation of the activity of the pathway (79). R-spondins (RSPOs) are secreted regulators of Wnt signaling and can enhance Wnt signaling. The RSPO2 gene encoding RSPOs has been confirmed to be an oncogene in colorectal cancer. Recent research has shown that RSPO2 is highly expressed in the CTNNB1 mutation subtype of HCC and can drive liver tumorigenesis by stimulating the activation of this pathway (80). Additionally, research indicates that various risk factors associated with HCC, including chronic HBV (81–85)or HCV (86–88) infection, Alcohol abuse (89, 90), Non-Alcoholic Fatty Liver Disease (NAFLD) (91, 92), and Aflatoxins Exposure (93, 94), can promote abnormal activation of this pathway through multiple mechanisms. This enhancement in the proliferation of affected hepatocytes and the overgrowth of adjacent normal hepatocytes can facilitate the progression of HCC precancerous lesions to HCC due to these factors.

### 4.2 Wnt/β-Catenin signaling pathway and HCC progression

#### 4.2.1 Cancer stem cells

Cancer stem cells (CSCs), also known as tumor-initiating cells (TICs), possess self-renewal and differentiation abilities comparable to normal stem cells, and are pivotal in the initiation, recurrence, and metastasis of tumors (95). The Wnt/β-catenin signaling pathway is one of the important ways to maintain the stemness of CSCs/TICs (96). Long non-coding RNAs (LncRNAs), an emerging regulatory factor, have been implicated in the development of cancer (97). The study found that LncTCF7 (98), Lnc-β-Catm (99), LncAPC (100), LncFZD6 (101), and LncTIC1 (102) exhibit high expression in HCC cells and liver CSCs/TICs. They contribute to the promotion of hepatic CSCs/TICs self-renewal by activating the Wnt/β-catenin signaling pathway. Additionally, several microRNAs (miRNAs), including miRNA-1246 (103), miRNA-5188 (104), miRNA-452 (105), miRNA-217 (106), and miRNA-HCC2 (107), also contribute to hepatic CSC stemness through activating this pathway. Furthermore, a functional read-through rt-circRNA named rtcisE2 was found to be highly expressed in liver TICs. It can also activate this pathway and promote the self-renewal of liver TICs, initiating the occurrence and metastasis of liver tumors (108).

Research indicates that Protein tyrosine kinase 2 (PTK2) stimulates the accumulation of β-catenin in the nucleus of HCC cells, thereby increasing Wnt/β-catenin signaling pathway activity, and in this way promoting stemness of CSCs and enhancing the tumorigenicity of HCC cells (109). Additionally, Sirtuin1 or Silent mating–type information regulation 2 homolog-1 (SIRT1) has likewise been shown to promote the activity of this pathway in hepatic CSCs by maintaining the stability of β-catenin, and in this way promotes CSC self-renewal (110). In fact, Mitogen-activated protein kinase 1 (MAPK1/MEK1) was previously found to promote the proliferation and self-renewal of hepatic stem cells by maintaining the stability of the SIRT1 protein, but that study did not investigate the mechanisms involved (111). Furthermore, FZD10 was shown to be substantially expressed in liver cancer CSCs by recent research. The pathway can be activated by its overexpression, which encourages the stemness of liver cancer CSCs and might be a new prognostic biomarker for HCC (112).

Tumor-associated macrophages (TAMs) are one of the primary subtypes of tumor-infiltrating immune cells, usually classified into M1 and M2 macrophages. The former generally plays an anti-tumor role, while the latter promotes tumor occurrence, metastasis, and angiogenesis through cytokine secretion, leading to tumor progression (113). Recent research found that M2 macrophages can secrete Tumor necrosis factor-α (TNF-α) and promote Epithelial-mesenchymal transition (EMT) of HCC and CSC stemness by inducing the Wnt/β-catenin signaling pathway (114). Additionally, Reactive oxygen species (ROS) overproduction has been reported to inhibit this pathway in HCC and reduce liver CSC stemness (115, 116). Glutaminase 1 (GLS1) is highly expressed in HCC, and GLS1 overexpression has been found to decrease ROS levels, reduce the inhibitory effect of ROS on the pathway and enhance CSC stemness (117). Furthermore, recent studies have discovered that Secretory clusterin (sCLU) may promote CSC stemness by activating the AKT/GSK3β/β-catenin axis (118). Moreover, this pathway can be activated by Ring finger protein 1 (Ring1), which is highly expressed in HCC, and in this way contributes to promoting the transformation of Hepatic progenitor cells (HPC) into CSC (119). In summary, this pathway has a crucial role in regulating CSC stemness, and more studies will be conducted in the future to reveal the mechanisms involved in this phenomenon.

#### 4.2.2 Proliferation, invasion, and metastasis of HCC

HCC cells exhibit strong capabilities in proliferation, invasion, and metastasis, which are important factors leading to poor prognosis of HCC patients (120). Numerous studies have shown that the Wnt/β-catenin signaling pathway plays an important role in regulating HCC cell proliferation, invasion and metastasis. Cripto-1 was found to be highly expressed in about 50% of HCC tissues (121). It can bind to the FZD7/LRP6 receptor and DVL3 and stabilize the expression of DVL3. In this way, it activates this pathway, which promotes the proliferation, invasion, and metastasis of HCC cells (122). Furthermore, the Tripartite motif (TRIM) protein family is reported to have extensive functions in tumor development, cell proliferation, and differentiation, although its effects vary across different tumors. Recent research has found that TRIM29 is downregulated in HCC tissue, potentially enhancing the activity of this pathway to promote the proliferation, invasion, and metastasis of HCC cells. Conversely, overexpression of TRIM29 inhibited this effect (123). However, this result is in stark contrast to previous studies, a discrepancy often attributable to genetic polymorphism and tumor complexity. Additionally, TRIM66 expression is upregulated in HCC compared to TRIM29. It can similarly activate this pathway and have the same effect on HCC cells (124). Similarly, overexpression of Ataxia telangiectasia group D complementing (ATDC) and Spindle and kinetochore-associated protein 2 (SKA2) in HCC similarly promotes HCC cell proliferation and invasion by activating this pathway (125, 126). Among the identified Wnts, Wnt7b has the ability to suppress Axin complex activity, stop β-catenin phosphorylation from being degraded, and facilitate its nuclear translocation, all of which contribute to the activation of the Wnt/β-catenin signaling pathway. It was found that TCP1 (also known as CCT1 subunit), which is overexpressed in HCC, can act as an upstream mediator of Wnt7b and increase Wnt7b expression, thus activating this pathway and enhancing the proliferation and metastasis of HCC cells (127). Furthermore, two deubiquitinating enzymes, USP9X and USP28, have significant expression in HCC and could similarly promote HCC cell proliferation by regulating the activity of this pathway (128, 129).

P62/IMP2, an oncofetal protein, was initially reported as a tumor-associated antigen in HCC. Research has established that p62/IMP2 is overexpressed in HCC tissues and may improve the invasion and metastasis capabilities of HCC by stimulating the Wnt/β-catenin signaling pathway (130). Additionally, Phosphatidylinositol 4-phosphate adaptor protein 2 (FAPP2) has also been identified as a tumor-associated regulatory factor related to tumorigenesis. It is reported to be highly expressed in HCC and can promote the proliferation and invasion of HCC cells by stimulating this pathway (131). SPINDOC (SPIN1 docking protein) and KIF18B (Kinesin family member 18B) have also been shown to be overexpressed in HCC and can similarly play a role in promoting the proliferative, invasive, and metastatic capacities of HCC cells through activation of this pathway (132, 133). ETS variant 4 (ETV4) is overexpressed in patients with HBV-related HCC and can activate the pathway, which promotes the proliferation, invasion, and metastasis of HCC cells, leading to the progression of HBV-associated HCC (134). Moreover, matrix metalloproteinases (MMPs) have been proven to be associated with HCC metastasis. Studies indicate that the expression of FBXO17 in HCC tissues was significantly higher than that in paracancerous tissues. It can promote HCC metastasis by down-regulating GSK-3β to mediate the activation of this pathway and increase the expression level of its downstream effector molecules (including MMP-2 and MMP-9) (135).

In addition, an increasing number of studies show that LncRNAs and miRNAs are equally involved in regulating this process and contributing to HCC progression. Research has discovered that LncRNA-miR194-2HG (136), LncRNA-DAW (137), LncRNA-NRAV (138), LncRNA-DUXAP10 (139), LncRNA-CRNDE (140), LncRNA OTUD6B-AS1 (141), and miR-550a-5p (142) are overexpression in HCC tissue. They can activate the Wnt/β-catenin signaling pathway to promote the proliferation, invasion, and metastasis of HCC cells. The inhibition of these genes’ expression may represent a viable therapeutic target for HCC.

#### 4.2.3 Epithelial-mesenchymal transition

EMT is a major factor in HCC cell invasion and metastasis and is induced by EMT-related transcription factor (EMT-TF). Elevated levels of vimentin and N-cadherin and decreased levels of E-cadherin are the main features of EMT (143). Several investigations have shown a robust connection between EMT and the Wnt/β-catenin signaling pathway. Recent studies have found Hepatic stellate cells (HSCs) can induce overexpression of miRNA-1246 in HCC, which can activate this pathway by suppressing the expression of its target gene, RORα, and in this way promote EMT (144). The trans-activation response DNA-binding protein of 43 kDa (TDP-43), a nuclear protein, is highly expressed in HCC tissues and activates the pathway by targeting inhibition of GSK3β expression to induce EMT (145). NFE2L3 (Nuclear factor erythroid 2-like 3) is a member of the CNC family of proteins and has been shown to be highly expressed in HCC. It also can induce EMT by activating this pathway (146). Furthermore, Rho guanine nucleotide exchange factor 11 (ARHGEF11), which is also overexpressed in HCC, can activate this pathway by increasing the nuclear translocation of β-catenin, thereby inducing EMT (147). Another study showed that the GBA1 protein, catalyzing the conversion of glucosylceramide (GlcCer) into ganglioside, is downregulated in HCC tissues and stimulates this signaling pathway by mediating GlcCer reprogramming, thus promoting EMT and enhancing the metastatic capability of HCC. Targeting the upregulation of GBA1 could be a potential therapeutic strategy against HCC metastasis in the future (148). Moreover, it has been reported that cysteine-rich protein 1 (CRP-1) is extensively expressed in various cancers, including HCC, and similarly induces EMT in a manner that activates this pathway (149).

#### 4.2.4 Glycolysis and angiogenesis

The proliferation, invasion, and metastasis of HCC cells is a complex process and requires a large amount of energy consumption, with glycolysis and angiogenesis being the main sources of energy in this process (150). Autophagy, as a programmed cell death mechanism, research has found that it can promote metastasis and glycolysis of HCC by increasing the expression of Monocarboxylate transporter 1 (MCT1) and activating the Wnt/β-catenin signaling pathway (151). As previously discussed, ROS produced by mitochondrial aerobic respiration can inhibit HCC progression by suppressing this pathway. Neoplastic cells opt for anaerobic glycolysis as their energy source, even when oxygen is present; this is referred to as the “Warburg effect” (152). Activation of the Wnt/β-catenin signaling pathway has been reported to stimulate the Warburg effect by up-regulating pyruvate dehydrogenase kinase isozyme 1 (PDK1), which promotes glycolysis in HCC cells, thereby supplying HCC cells with energy and enhancing HCC cell proliferation, invasion, and metastasis. Peroxisome proliferator-activated receptor-gamma (PPARγ) co-activator-1α (PGC-1α), a tumor suppressor, participates in cancer pathogenesis, progression, and metabolism. Based on research findings, it has been observed that PGC-1α inhibits the PDK1 pathway, thereby decreasing PDK1 expression and subsequently impeding the metastasis of HCC (153). Thus, the downregulation of PGC-1α expression in HCC may promote the Warburg effect and energize HCC progression by stimulating this pathway activity. Additionally, a recent study discovered that Galectin-3 is involved in HCC metastasis and activates this pathway by inducing Phosphatidylinositol 3-kinase (PI3K)/Akt axis-mediated degradation of GSK-3β. Finally, the β-catenin/TCF4 transcriptional complex directly targets IGFBP3 and waveform proteins, thereby promoting angiogenesis and EMT in HCC (154).

#### 4.2.5 Hypoxia

Hypoxia, a common feature of all solid tumors, results from an imbalance between oxygen supply and consumption in proliferative tumors. It is essential for the occurrence and progression of tumors (155). Like most solid tumors, HCC also exhibits a hypoxic microenvironment. Abundant evidence indicates that there is crosstalk between hypoxia-inducible factor hypoxia-inducible factor (HIF) and the Wnt/β-catenin signaling pathway and that it could contribute to the development of HCC (156–158). It has been found that hypoxia causes β-catenin to be expressed and accumulate in HCC cell lines, which facilitates invasion and metastasis (159). Further studies showed that crosstalk between hypoxia and this pathway is mediated through HIF-1α (160). However, the mechanisms involved remain unclear. BCL9, an essential co-activator of this pathway, is discovered to be overexpressed in HCC. It was found that hypoxia may cause BCL9 to be overexpressed in HCC via HIF-1α, activating this pathway and accelerating the growth, metastasis, and angiogenesis of HCC cells (156). Furthermore, this study provides evidence for the crosstalk between this pathway and the hypoxic and demonstrates that the specific regulation of BCL9 by HIF-1α may be a potential crosstalk mechanism between the two.

## 5 Role of Wnt/β catenin signaling pathway in HCC therapy resistance

Overcoming multidrug resistance (MDR) poses a substantial challenge in the management of hepatocellular carcinoma (HCC), among other malignancies, where it has emerged as a key obstacle. An important factor in MDR is the overexpression of ATP-binding cassette (ABC) transporters, which facilitates the expulsion of antitumor drugs from cells, thereby preventing their accumulation intracellularly and mitigating their cytotoxicity (161). It is reported that the Wnt/β-catenin signaling pathway can regulate tumor therapy resistance by modulating the expression of ABC transporters (162). FZD7 as an FZD receptor, ABC transporters (ABCB1, ABCC1, and ABCC2) can be upregulated in HCC cells by FZD7 overexpression via this pathway which results in increased therapy resistance in HCC. Quercetin can reverse this effect and enhance the drug sensitivity of HCC (163).

Additionally, a substantial amount of data indicates that this pathway is crucial in mediating chemoresistance in hepatocellular carcinoma. Gankyrin has been shown to be overexpressed in a variety of cancers. In HCC, it can activate this pathway to upregulate the expression of its target gene c-Myc, inducing metabolic reprogramming in HCC cells, and thus promoting HCC tumorigenesis, metastasis, and therapy resistance. Inhibiting c-Myc expression might be an optimal treatment strategy for HCC patients with high Gankyrin expression (164). As mentioned earlier, PROX1 is a specific gene in liver development. Studies have found that PROX1 is highly expressed in HCC, and it can activate this pathway by stimulating β-catenin transcription, promoting HCC cell proliferation and sorafenib resistance (165). Additionally, NIMA-related kinase 2 (Nek2) and LRP8 expression were found to be upregulated in HCC and contribute to sorafenib resistance in HCC by the same mechanism (166, 167). Recent studies have found that Src homolog and collagen homolog 3 (Shc3) are overexpressed in chemotherapy-resistant HCC and similarly activate this pathway, causing resistance to sorafenib and doxorubicin in HCC (168). Moreover, FZD10, found to be overexpressed in liver CSCs, activates this pathway to promote the resistance to Lenvatinib of HCC. Targeted knockdown of FZD10 can restore the sensitivity of lenvatinib-resistant HCC to lenvatinib (112). Furthermore, research has demonstrated that cisplatin-resistant HCC has substantially increased levels of miR-130a. Specifically, its overexpression inhibits the tumor suppressor gene RUNX3, which in turn activates this pathway and augments the HCC’s resistance to cisplatin. Knockdown of miR-130a can reverse the resistance of HCC to cisplatin (169). Additionally, the activation of this pathway is also facilitated by the overexpression of Krüppel-like factor 8 (KLF8) in HCC. This ultimately increases the chemoresistance of HCC to sorafenib and cisplatin. Compared to control HCC cells, the knockdown of KLF8 can significantly increase chemosensitivity (170). DVL1, an important component of the Wnt/β-catenin signaling pathway, stabilizes β-catenin activity and mediates Wnt signaling. DVL1 expression was found to be overexpressed in 5-FU-resistant HCC cells and may enhance HCC resistance to 5-FU by activating the Wnt/β-catenin signaling pathway (171).

Due to the high expression of various drug-resistant genes, HCC patients are often insensitive to chemotherapy. Consequently, for HCC patients who have lost the opportunity for surgery, radiation therapy has gradually become an important treatment method. However, some HCC patients still show resistance to radiation therapy. Mesenchymal stem cells (MSCs), as a crucial component of the tumor microenvironment, have been proven to participate in tumor therapy resistance (172). Research has found that Irradiated MSCs (IR-MSCs) can promote the maintenance of CSC stemness by activating the Wnt/β-catenin signaling pathway, leading to radiotherapy resistance in HCC (173). This suggests that the activation of this pathway under radiotherapy conditions might be responsible for this resistance.

In addition, with the continual advancements in HCC treatment modalities, immunotherapy has progressively emerged as a pivotal therapeutic approach. However, there is considerable variation in the response of HCC patients to immunotherapy. It is reported that cancer immune evasion and resistance to Immune checkpoint inhibitors (ICIs) are mediated by the Wnt/β-catenin signaling system (174). Research has found that in HCC, the activation of this pathway can compromise dendritic cell recruitment and reduce T cell activity, promoting immune evasion in HCC cells and inducing resistance to ICI drugs like PD-1 (Programmed cell death 1) (175). In syngeneic mouse models, using the chemically optimized RNAi trigger drug DCR-BCAT, targeting the CTNNB1 gene encoding β-catenin, it was found that DCR-BCAT could significantly increase T cell infiltration and enhance tumor sensitivity to ICIs (176). Additionally, a study using a biological nanoparticle delivery method delivered small interfering RNA (siRNA) targeting β-catenin directly into Extracellular vesicles (EVs), resulting in not only reduced growth of HCC cells but also enhanced responsiveness to PD-1 treatment (177). Combining these studies, it is possible to deduce that the Wnt/β-catenin signaling pathway significantly influences the immune evasion mechanism of HCC cells. Potentially, inhibiting this pathway could improve the efficacy of HCC immunotherapy.

## 6 Potential role of Wnt/β catenin signaling pathway in HCC targeted therapy

The incidence of HCC is increasing every year, with only a small proportion of patients eligible for surgical resection. Chemotherapy is the leading therapeutic approach for HCC patients who do not qualify for surgical resection. Significant advancements have been achieved in molecular targeted therapy in recent years, and the survival rate of HCC patients has been significantly increased through the combination of targeted therapy and chemotherapy. Given the important role of the Wnt/β-catenin signaling pathway in HCC tumorigenesis, progression, and therapy resistance, targeting this pathway may be a new potential therapeutic approach for HCC patients.

Numerous pharmaceuticals that inhibit this pathway have been developed as a result of years of research on this pathway. These drugs include monoclonal antibodies targeting Wnts and FZD receptors, molecules inhibiting their secretion and interaction, and small molecule inhibitors that stabilize the β-catenin destruction complex and block the binding of β-catenin to specific transcription co-activators (**Table 1**). Additionally, Glypican-3 (GPC3), a Heparan sulfate proteoglycan (HSPG) that is overexpressed in HCC, can recruit Wnts to the cell surface and stimulate cell proliferation. A monoclonal antibody targeting GPC3, HS20, has been reported, which can inhibit this pathway in HCC cells and exerts a potent antitumor effect by targeting GPC3 (189). Furthermore, several other drugs commonly used in clinical practice have been proven to have anti-Wnt/β-catenin signaling pathway activity, including indomethacin, pyrvinium, sulindac, aspirin, celecoxib, rofecoxib, peruvoside, and pirfenidone (183, 190, 191). However, whether these drugs have anti-tumor efficacy has not yet been determined in clinical settings.

**Table 1:** Drugs that inhibit Wnt/β-catenin signaling pathway.

Drug	Classification	Intervention mechanism	References
Anti-Wnt-2 monoclonal antibody	Wnts monoclonal antibody	Targets Wnt2 and inhibits the activation of the Wnt/β-catenin signaling pathway induced by it	(178)
OMP-18R5	FZD monoclonal antibody	Targeting the FZD receptor and thereby inhibiting the Wnt/β-catenin signaling pathway	(179)
LGK-974/ETC-159/CGX1321/RXC004	PORCN inhibitor	Blocking Wnt secretion by inhibiting PORCN, thereby inhibiting the Wnt/β-catenin signaling pathway and thus suppressing tumor cell growth	(180)
OMP-54F28	FZD8 decoy receptor	Acts as a decoy receptor for FZD8 and can inhibit the Wnt/β-catenin signaling pathway by isolating the Wnt ligand from competitive binding to the FZD8 receptor	(181)
Salinomycin	LRP5/6 inhibitor	Inhibits the Wnt/β-catenin signaling pathway by preventing β-catenin from binding to it through inhibition of LRP5/6	(182)
XAV939/G007-LK/G244-LM/RK-287107/JW55/K-756/IWR-1/MSC2504877/AZ1366/JW74/NVP-TNKS656	Tankyrase (TNKS) inhibitors	Stabilization of AXIN protein by inhibiting TANKS-mediated AXIN degradation, which increases β-catenin destruction complex activity and β-catenin phosphorylation, leading to inhibition of the Wnt/β-catenin signaling pathway	(183)
PKF118-310/PKF115-584/CGP049090	β-catenin/TCF antagonist	Inhibition of β-catenin binding to TCF decreases c-Myc, cyclinD1, and survivin, target genes of the Wnt/β-catenin signaling pathway, thus exerting antitumor effects	(184)
PRI-724	CBP/β-catenin antagonist	Acts by inhibiting β-catenin binding to the coactivator CAMP-response element binding (CREB)-binding protein (CBP)	(185)
IGG-001	CBP antagonist	Prevents β-catenin from acting in conjunction with CPB and inhibits tumor growth by competitively binding CBP	(186)
IC-2	Novel derivatives of IGG-001	Like IGG-001, IC-2 acts by inhibiting the binding of β-catenin to CPB, and in this way inhibits hepatic CSC stemness	(187)
DKN-01	DKK1 monoclonal antibody	Dickkopf-1 (DKK1), a regulator of the Wnt signaling pathway, is overexpressed in a variety of cancers and has been associated with tumor immunosuppression, and DKN-01 enhances the innate immune response of tumors by blocking the action of DDK1	(188)

Several inhibitors or modulators of this pathway are currently in clinical trials. CGX1321, a PORCN inhibitor, has been tested in phase I clinical trials in patients with HCC and Cholangiocarcinomas (CCA) (NCT03507998). OMP-18R5, a novel monoclonal antibody against FZD that can target the FZD receptor and thereby block the Wnt/β-catenin signaling pathway, has been evaluated for efficacy and safety in a clinical trial in relevant solid tumors (NCT01345201). In addition, OMP-54F28, an FZD8 decoy receptor, can competitively bind to Wnts to block this pathway, and its efficacy in combination with sorafenib was tested in patients with advanced HCC in a phase I clinical trial (NCT02069145). Dickkopf-1 (DKK1), a secreted regulator of the Wnt signaling pathway, is overexpressed in a variety of cancers and has been associated with tumor immunosuppression, and DKN-01 can act by blocking DKK1 and in so doing enhances the innate immune response of tumors. A clinical trial (NCT02375880) evaluated the clinical value of DKN-01 in combination with gemcitabine and cisplatin in the treatment of patients with biliary tract cancer, followed by another clinical trial (NCT03645980) evaluating the antitumor activity and safety of DKN-01 in combination with sorafenib in patients with advanced HCC. In addition, PRI-724, a Wnt signaling pathway inhibitor, has demonstrated its efficacy and safety in an earlier solid tumor clinical trial (NCT01302405), but no clinical trial has yet evaluated its clinical value in the treatment of HCC patients. Although several drugs have been shown to have antitumor activity in preclinical models of HCC, most of them have not yet entered clinical trials, and more clinical trials are still needed to evaluate their efficacy and safety in future studies.

Diverse natural bioactive compounds derived from various dietary sources have been found to inhibit this pathway and demonstrate antitumor properties in HCC, according to recent research (**Table 2**). Compared to conventional chemotherapy drugs, these natural bioactive compounds have lower toxicity and are easily obtainable through diet, making them excellent adjuvant anti-cancer agents. However, it is still unclear if these chemicals can efficiently reach the tumor site and exert antitumor effects because the majority of them are derived from plants and have low bioavailability. Moreover, the majority of naturally occurring bioactive substances frequently influence additional molecular pathways in addition to this pathway. Future research should focus on addressing these issues.

**Table 2:** Natural bioactive compounds that inhibit Wnt/β-catenin signaling pathway.

Name	Origin	Intervention mechanism	References
Astaxanthin (ASX)	Microorganisms and marine animals	Blocking GSK-3β phosphorylation and degradation by inactivating the PI3K/Akt axis, thereby inhibiting the Wnt/β-catenin signaling pathway and exerting inhibition of HCC cell proliferation and induction of apoptosis	(192)
Morin	Almond hulls and old fustic	Inhibition of Wnt/β-catenin signaling pathway by inhibiting Mammalian Sterile 20-like Kinase 1 (Mst1) overexpression promotes apoptosis in HCC cells	(193)
6-C-(E-Phenylethenyl)(6-CEPN)	Tomato and citrus fruits	Inhibition of the Wnt/β-catenin signaling pathway by up-regulating GSK-3β expression induces β-catenin degradation and inhibits its nuclear translocation, attenuating HCC cell stemness	(194)
Swertiamarin(STM)	Gentianaceae plants	Inhibition of the Wnt/β-catenin signaling pathway through down-regulation of FRAT1 exerts an inhibitory effect on proliferation, metastasis, and invasion of HCC cells	(195)
Berberine(BBR)	Herbal plants	Antagonizing the Wnt/β-catenin signaling pathway by inhibiting β-catenin translation	(196)
Daucosterol	Plants	Inhibits the Wnt/β-catenin signaling pathway by down-regulating β-catenin expression and exerts inhibitory effects on proliferation, metastasis, and invasion of HCC cells	(197)
Daphnetin	Genus Daphne	Promoting apoptosis in HCC cells by inhibiting the Wnt/β-catenin signaling pathway	(198)
Emodin	Rheum palmatum, Polygonum cuspidatum and Polygonum multiflorum	Inhibition of the Wnt/β-catenin signaling pathway and inhibition of HCC cell invasion and metastasis by inducing β-catenin degradation	(199)
Curcumin	Curcuma	Inhibition of HCC invasion and EMT by regulating the TET1/Wnt/β-catenin signaling pathway	(200)
Gynura divaricata	The aerial part of G. divaricate/hepatitis grass	Inhibition of hepatic CSCs growth and prolongation of the antitumor statute of limitations of cisplatin through inhibition of the Wnt/β-catenin signaling pathway	(201)
Broussochalcone A (BCA)	Broussonetia papyrifera	Inhibition of the Wnt/β-catenin signaling pathway by promoting phosphorylation/ubiquitin-dependent degradation of β-catenin reduces HCC cell viability	(202)
Strophanthidin	Strophanthus kombe	Anti-tumor activity through inhibition of MAPK, PI1K/AKT/mTOR and Wnt/β-catenin signaling pathways	(203)
Dendrobium candidum extract (DCE)	Dendrobium	Blocking the Wnt/β-catenin signaling pathway by decreasing β-catenin levels and regulating its downstream target genes inhibits HCC cell proliferation and induces apoptosis	(204)
Alpha-linolenic acid	Vegetable oil	Suppression of Wnt/β-catenin signaling pathway and inhibition of HCC cell growth by up-regulation of FXR expression	(205)
Tetrandrine	Stephania tetrandra S. Moore	Regulation of HCC cell metastasis by inhibiting Wnt/β-catenin signaling pathway activity and decreasing Metastatic tumor antigen 1 (MTA1) expression	(206)
Polysaccharides	Hemerocallis citrina Baroni	HCC cell cycle arrest and apoptosis through inhibition of the Wnt/β-catenin pathway	(207)

In addition, some bioactive molecules have been demonstrated to inhibit this pathway and exert anti-tumor effects (**Table 3**). Several anesthetics commonly used in clinical settings have been found to exhibit antitumor properties through the inhibition of the Wnt/β-catenin signaling pathway. Sevoflurane (Sevo), an inhalational anesthetic, has been demonstrated to inhibit tumor growth of HCC (239). Mechanistic studies indicate that it can regulate the PTEN/Akt/GSK-3β/β-catenin axis by down-regulating miR-25-3p expression, inhibit the Wnt/β-catenin signaling pathway, and exert anti-tumor effects (240). Like Sevo, propofol is an intravenous anesthetic commonly used in surgery, and it similarly inhibits this pathway, exerting an inhibitory effect on the growth and invasion of HCC cells (241, 242).

**Table 3:** Bioactive molecules inhibiting Wnt/β-catenin signaling pathway.

Name	Classification	Intervention mechanism	References
MiR-143HG	Long non-coding RNAs	MiR-143HG mediates elevated APC expression by inhibiting miR-155 expression, which promotes β-catenin phosphorylation degradation, inhibits the Wnt/β-catenin signaling pathway, and suppresses HCC cell proliferation and metastasis	(208)
MiR-300	MicroRNA	MiR-300 inhibits the Wnt/β-catenin signaling pathway and suppresses HCC cell growth by down-regulating the expression level of its target gene CREPT	(209)
MiR-320a	MicroRNA	MiR-320a can directly target β-catenin and inhibit the Wnt/β-catenin signaling pathway by down-regulating the expression of β-catenin and its target genes to inhibit HCC cell proliferation	(210)
TRIM29/TRIM36	Tripartite motif family (TRIM) proteins	Both inhibit the enhancement, invasion, and metastasis of HCC cells by suppressing the Wnt/β-catenin signaling pathway	(123, 211)
PGC-1α	Coactivator	PGC-1α inhibits the Warburg effect by suppressing the WNT/β-catenin/PDK1 axis, thereby suppressing HCC cell invasion and metastasis	(153)
C12orf75	DNA	Downregulation of the C12orf75 gene inhibits metastasis and invasion of HCC cells by suppressing the Wnt/β-catenin signaling pathway	(212)
LIM Homeobox-2(LHX2)	Transcriptional factor	LHX2 mediates the breakdown of the β-catenin/TCF4 complex and induces the expression of multiple Wnt inhibitors, leading to a Wnt/β-catenin signaling pathway that inhibits HCC cell growth	(213)
LHX6	Transcriptional factor	LHX6 inhibits Wnt/β-catenin and P53 signaling pathways induced by Microcystin-LR in HCC to suppress proliferation, invasion, and metastasis of HCC cells	(214)
SOX11	Transcriptional factor	SOX11 prevents TCF4 from binding to β-catenin by increasing its phosphorylation, leading to a decrease in the activity of the Wnt/β-catenin signaling pathway, which inhibits the growth of HCC cells and induces apoptosis	(215)
Cepharanthine hydrochloride(CH)	Compound	CH can inhibit HCC cell proliferation and invasion and induce apoptosis by inhibiting Wnt/β-catenin signaling	(216)
Large tumor suppressor kinase 2 (LATS2)	Suppressor protein	LATS2 upregulates DRP1 expression through the Wnt/β-catenin signaling pathway, leading to increased mitochondrial fragmentation and thus promoting HCC cell death	(217)
FH535/FH535-N	Inhibitor	FH535 and its derivative FH535-N exert antitumor effects by inhibiting autophagic flux in HCC cells through regulating the Wnt/β-catenin signaling pathway, and the combination with sorafenib can increase efficacy	(218–220)
Klotho	Tumor suppressor gene	Klotho overexpression inhibits HCC progression and induces apoptosis by negatively regulating the Wnt/β-catenin signaling pathway	(221)
S-Adenosylmethionine (SAMe)	Amino acid	SAMe and its metabolite methylthioadenosine (MTA) can inhibit the Wnt/β-catenin signaling pathway in HCC through several mechanisms	(222)
H2S	Compound	H2S inhibits H2O2-induced HCC proliferation and metastasis by modulating the Wnt/β-catenin signaling pathway	(223)
MiR-329-3p	MicroRNA	MiR-329-3p inhibits the proliferation and metastasis of HCC cells by suppressing USP2-mediated activation of the Wnt/β-Catenin pathway	(224)
MiR-639	MicroRNA	MiR-639 inhibits the proliferation and metastasis of human hepatocellular carcinoma cells by down-regulating the KAT7/Wnt/β-catenin signaling pathway	(225)
MiR-212	MicroRNA	MiR-212 inhibits HCC cell growth through the Wnt/β-catenin signaling pathway	(226)
Combretastatin A-1 phosphate (CA1P)	Inhibitor	CA1P inhibits the Wnt/β-catenin signaling pathway and exerts antitumor activity by inducing AKT inactivation, leading to GSK-3β activation	(227)
Trans-chalcone (TC)	Compound	TC mediates cellular autophagy and induces HCC cell death through p53 up-regulation and β-collagen down-regulation	(228)
HYD-PEP06	Polypeptide	HYD-PEP06 inhibits HCC metastasis, EMT, and CSC stemness by suppressing the activation of PI3K/AKT and Wnt/β-catenin signaling pathways.	(229)
Homoharringtonine (HHT)	Compound	HHT inhibits the proliferation and metastasis of HCC cells by inducing EphB4 inhibition that promotes phosphorylation and loss of β-linker proteins	(230)
Secalonic acid-F(SAF)	Mycotoxin	SAF can inhibit HCC progression by targeting MARCH 1 and regulating the PI3K/AKT/β-catenin signaling pathway	(231)
Prohibitin1 (PHB1)	Mitochondrial chaperone protein	PHB1 acts as a negative regulator of the Wnt/β-catenin signaling pathway and exerts antitumor effects by inhibiting the Wnt/β-catenin signaling pathway	(232)
Cellular retinol binding protein-1(CRBP-1)	Protein	CRBP-1 suppresses CSC stemness by inhibiting the Wnt/β-catenin signaling pathway	(233)
Astrotactin 1 (ASTN1)	Protein	ASTN1 can inhibit the metastatic and invasive ability of HCC by suppressing the Wnt/β-catenin signaling pathway	(234)
P7 trans-regulated protein 3 (P7TP3)	Protein	P5TP7 regulates HCC proliferation, invasion, metastasis, adhesion, and cell cycle progression through the Wnt/β-catenin signaling pathway, thereby inhibiting HCC	(235)
Aquaporin 9 (AQP9)	Protein	AQP9 overexpression reduces β-catenin levels in HCC cells and inhibits HCC cell growth and metastasis via the Wnt/β-catenin signaling pathway	(236)
DDX5	RNA Helicase	DDX5 is a negative regulator of the Wnt/β-catenin signaling pathway in HBV-associated HCC and is lowly expressed in HBV-associated HCC. miR17-92/miR106b-25 restored the expression level of DDX5 in HBV-associated HCC	(237)
Glutathione S-transferase Zeta 1-1 (GSTZ1-1)	Transferase	GSTZ1-1 may act as a tumor suppressor by inhibiting Wnt/β-catenin signaling pathway activity in HCC cells	(238)

siRNA and antisense RNA are currently the most commonly used genetic tools, known for their specificity and ease of operation. They treat diseases caused by gene mutations or overexpression by reducing the expression of target genes and have been widely used in cancer therapy (243–245). Gene therapy based on siRNA or antisense RNA is considered another method to inhibit the Wnt/β-catenin signaling pathway. Studies have utilized siRNA targeting β-catenin to explore its value in HCC treatment. siRNA-CTNNB1 targeting to reduce β-catenin expression can inhibit this pathway and decrease the production of target genes cyclin-D1 and GS, impairing the proliferation and survival of HCC cells (246). Another study demonstrated that using siRNA to target β-catenin expression could arrest tumor cells in the G0/G1 phase of the cell cycle, thus inhibiting HCC cell proliferation (247). Furthermore, a CTNNB1 mutant mouse HCC model induced by Phenobarbital (PB) and Diethylnitrosamine (DEN) demonstrated that inhibiting β-catenin expression with locked nucleic acid (LNA) antisense oligonucleotides resulted in decreased HCC cell proliferation and increased apoptosis. In contrast, this effect was not observed in a rodent HCC model lacking CTNNB1 (248).

Existing studies have shown the importance of targeting Wnt/β-catenin signaling pathway transduction in the treatment of HCC, and a large number of preclinical studies have provided sufficient evidence for this. Various therapeutic strategies targeting this pathway have been developed. Nevertheless, due to the complex function of this pathway in the human body, these inhibitors exert anti-tumor activity while also inhibiting the Wnt/β-catenin signaling pathway in other normal tissues, resulting in toxic effects on normal tissues. This has severely limited the development of current therapeutic strategies targeting this pathway.

## 7 Conclusions and prospects

The role of the Wnt/β-catenin signaling pathway in the tumorigenesis, progression, and therapy resistance of HCC is indisputable. Targeting this pathway is an attractive target in HCC treatment. Increasingly studies have proven that drug or molecular targeting can block this pathway, which ultimately reduces tumor growth and improves therapeutic efficacy. With the increasing research on the Wnt/β-catenin signaling pathway, various inhibitors have been developed that can target this pathway. Unfortunately, owing to the toxicology of these inhibitors, there aren’t any authorized medications for the clinical therapy of HCC at this time.

In conclusion, targeting the Wnt/β-catenin signaling pathway still remains a significant challenge. Future studies should further deepen our understanding of the regulatory mechanisms of this pathway in HCC and guide the development of new HCC-targeted therapeutic strategies. Additionally, the development of specific targeted drugs that can selectively inhibit this pathway in tumor tissues remains a focus of future research.

## Author contributions

ZKZ: Writing – original draft, Writing – review & editing. TC: Software, Writing – review & editing. FW: Writing – review & editing. ZMZ: Writing – review & editing. YS: Writing – review & editing. CG: Supervision, Writing – review & editing. XX: Funding acquisition, Writing – review & editing. HZ: Writing – review & editing.
